# Gecko‐Like Multi‐Directional High‐Power Magneto–Mechano–Electric Energy Harvesters for Self‐Powered, Hundred‐Meter‐Scale LoRa Communication

**DOI:** 10.1002/advs.74974

**Published:** 2026-03-28

**Authors:** Shitong Fang, Haoxian Peng, Mingjing Cai, Liuchao Jin, Yuelong Yu, Mianxin Xiao, Qingyang Xu, Jiacheng Hou, Degui Yu, Zhihui Lai, Xin Li, Shuxiang Dong, Biao Wang, Wei‐Hsin Liao

**Affiliations:** ^1^ Guangdong Key Laboratory of Electromagnetic Control and Intelligent Robots, College of Mechatronics and Control Engineering Shenzhen University Shenzhen China; ^2^ National Key Laboratory of Green and Long‐Life Road Engineering in Extreme Environment (Shenzhen) Shenzhen University Shenzhen China; ^3^ Guangzhou Institute of Technology and School of Mechano‐Electronic Engineering Xidian University Guangzhou China; ^4^ Department of Mechanical and Automation Engineering The Chinese University of Hong Kong Hong Kong China; ^5^ Institute of Artificial Intelligence, School of Future Technology Shanghai University Shanghai China; ^6^ Department of Medical Ultrasound, West China Hospital Sichuan University Chengdu China; ^7^ Institute of Intelligent Design and Manufacturing The Chinese University of Hong Kong Hong Kong China

**Keywords:** energy harvesting, LoRa communication, magneto–mechano–electric, multi‐directional, self‐powered

## Abstract

Magneto–mechano–electric energy harvesting from stray magnetic fields near power infrastructure is a promising approach for autonomous wireless sensor networks. However, state‐of‐the‐art magneto–mechano–electric energy harvesters (MME‐EHs) fail to capture multi‐directional 50/60 Hz magnetic fields of power infrastructure due to frequency mismatch and unidirectional responsiveness. To break this bottleneck, this study proposes a gecko‐like MME‐EH that mimics the flexible, symmetrical movement of gecko limbs to achieve coordinated twisting and bending. The centrally symmetric configuration with low clamping loss allows for high magnetic loads and large beam deformation, resulting in high energy output for driving long‐distance wireless sensor systems. Under a weak 60 Hz magnetic field ($H_{\text{ac}}$ = 1 Oe), the bio‐inspired multi‐directional MME‐EH generates average power of 12.8, 0.24, 0.15 ${\mathrm{mW}}_{\mathrm{RMS}}$ in x‐, y‐, and z‐directions, respectively, representing power output and power density improvements of up to 17.75 and 5 times, respectively, over state‐of‐the‐art results. The application tests show that the proposed harvester can successfully achieve self‐powered LoRa communication over 300 meters outdoors, scaling from the conventional meter scale to an unprecedented hundred‐meter scale in the MME area. Furthermore, the long‐distance operational status monitoring of the transformer is demonstrated by coupling the proposed MME‐EH with the LoRa communication system.

## Graphical abstract

Gecko like multi directional high power magneto mechano electric energy harvester (MME EH) for self powered, hundred meter scale LoRa communication. Inspired by the symmetric and flexible limb motion of geckos, the biomimetic harvester enables coordinated bending twisting responses to weak, low frequency (50/60 Hz) stray magnetic fields from power infrastructure in three dimensions. The harvested magnetic energy is efficiently converted into electrical power to drive long range LoRa wireless sensing, enabling battery free, real time condition monitoring of transformers and power grid systems.

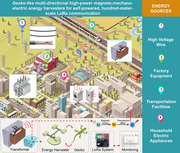

## 1 Introduction

With the rapid development of the Power Internet of Things (PIoT), a large number of battery‐powered wireless sensors and communication devices face huge economic issues due to the frequent maintenance and replacement [1, 2, 3]. Besides, the depletion of batteries will disrupt the monitoring system, impairing the ability to promptly address potential hazards [4, 5, 6, 7]. Powering a large number of sensors and communication units through a conventional electric network is impractical, as it necessitates numerous power interfaces and voltage conversion components [8, 9]. Consequently, providing sustainable energy sources for PIoT operation remains a fundamental challenge, particularly in achieving real‐time monitoring of power infrastructure.

To address these challenges, researchers have proposed to harvest the vibration energy [10, 11, 12], wave energy [13, 14, 15], solar energy [16, 17, 18], and magnetic field energy [19, 20, 21, 22] for powering PIoT devices. Among these, harvesting the 50/60 Hz magnetic field energy around power infrastructure appears to be promising due to its stability and sustainability. For the remote power infrastructure, the long‐distance communication systems are often essential. Whereas these magnetic fields around power infrastructure are normally weak with field strength lower than 1 Oe, making it challenging to design an energy harvester that can support long‐range communication systems with sensors such as Long Range Radio (LoRa) [23, 24, 25, 26] and SigFox. In previous applications, coil‐based devices have typically been used to scavenge magnetic field energy in the environment. However, in low‐frequency stray magnetic fields, these devices can hardly achieve efficient magnetic–electric conversion, which is therefore inadequate for powering long‐range communication systems [27, 28, 29, 30].

The emerging magneto–mechano–electric energy harvesters (MME‐EHs) [31, 32, 33, 34] are considered as a promising alternative for collecting waste magnetic field energy due to their compact size and high output power. Early MME‐EHs [35, 36] commonly adopted cantilever beam structures, where piezoelectric composite materials were integrated with permanent magnets attached to their free ends. Their working principle is as follows: when an external alternating magnetic field ($H_{\text{ac}}$) excites the system, the permanent magnet generates magnetic torque. This torque is then transferred through elastic coupling to the piezoelectric layer, resulting in electrical output across the connected load circuit. To further increase the power output of the MME‐EHs, Ryu et al. [37] introduced magnetostrictive materials to realize a strong magnetoelectric coupling effect. Lee et al. [3] proposed a hybrid system with a piezoelectric MME generator, an electromagnetic induction coil, and a triboelectric nanogenerator. Yu et al. [38] proposed a symmetrically coupled dual‐mode tuning fork structure, which delivers an output power 437% higher than the traditional single cantilever beam structure. There was also a bionic dragonfly MME‐EHs proposed by Chang et al. [39], whose working principle is based on the flapping behavior of dragonflies during flight as well as the phase difference between the front and rear wings. This structure operates in a cross‐antisymmetric bending mode with strong MME coupling, achieving an output power up to 4.45 ${\mathrm{mW}}_{\mathrm{RMS}}$ under a weak AC magnetic field of 1 Oe.

**FIGURE 1:**
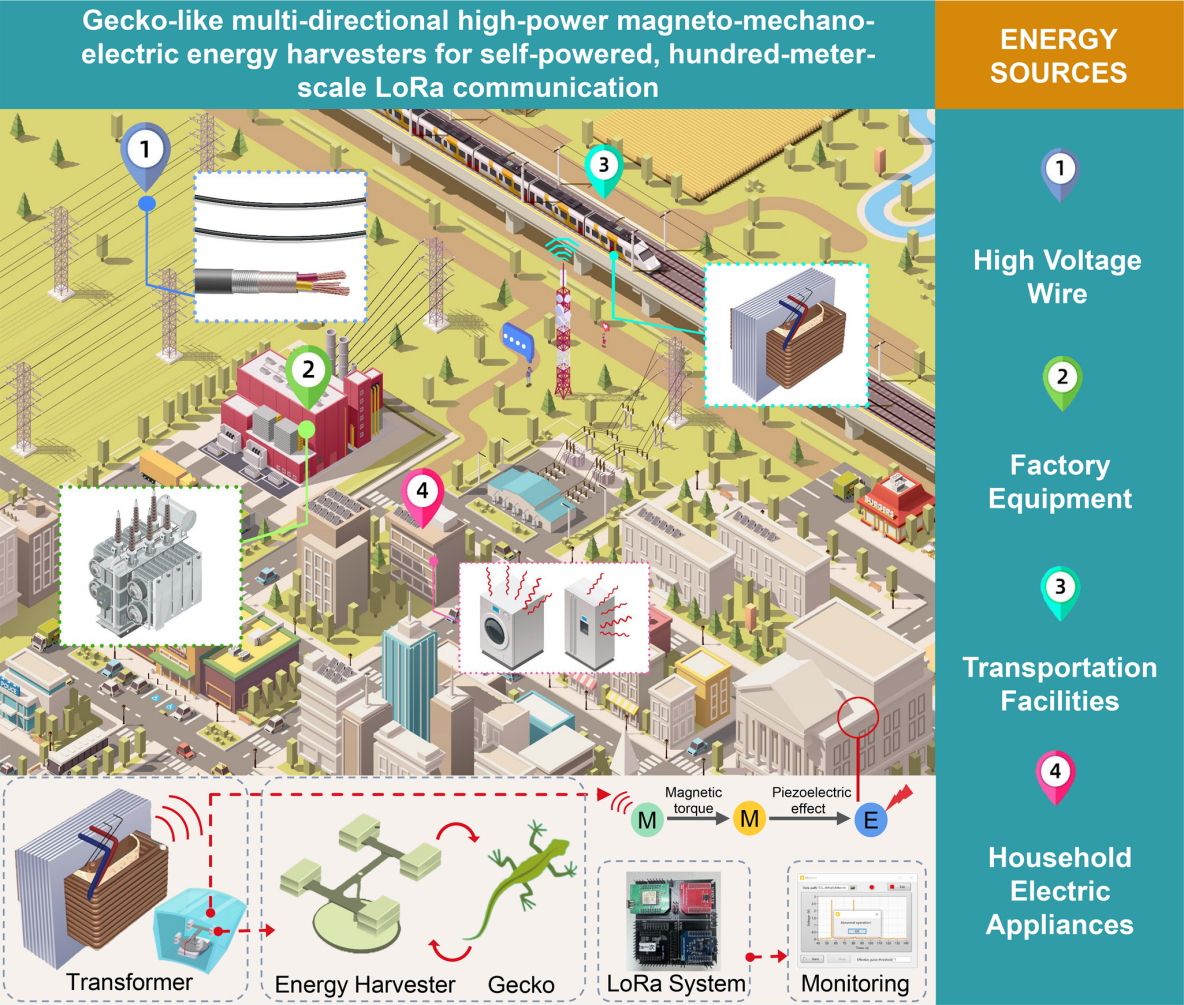
Application schematic of the proposed gecko‐like MME‐EH for the self‐powered, hundred‐meter‐scale LoRa communication.

In practice, the magnetic fields emitted by various power grid systems and machine equipment in factories, transportation facilities, and household appliances are randomly distributed in space, as shown in Figure 1. Transformers and high voltage wires are the typical power infrastructure that often generate leakage and multi‐directional magnetic fields. Effectively harvesting the magnetic energy could drive the sensor‐based communication systems, enabling operational status monitoring of power infrastructure. However, the MME‐EHs introduced above can only respond to the magnetic field direction along their length direction, deteriorating energy harvesting efficiency during operation. To alleviate this issue, Yu et al. [40] proposed an X‐shaped MME‐EH with a fourfold architecture and symmetrically arranged magnets at each end, facilitating near‐isotropic omnidirectional energy harvesting in the in‐plane direction. Results show that the output power under *x*‐ and *y*‐directional magnetic fields of 1 Oe is 1.72 ${\mathrm{mW}}_{\mathrm{RMS}}$ for each orientation. However, it cannot harvest the *z*‐directional magnetic field energy. Similarly, Yu et al. [41] reported a T‐shaped MME‐EH for capturing energy from tridimensional (3D) magnetic fields by generating multiple modal responses of beams. Under an *x*‐direction magnetic field of 1 Oe, the output power is up to 4.02 ${\mathrm{mW}}_{\mathrm{RMS}}$. However, its power output under *z*‐directional magnetic field is as low as 0.008 ${\mathrm{mW}}_{\mathrm{RMS}}$ due to the frequency mismatch. Currently, few studies have reported MME‐EHs that can indeed resonate at 50/60 Hz under 3D magnetic fields, limiting their real‐world applications. Furthermore, the energy of MME‐EHs harvested from the ultra‐low‐intensity ($H_{\text{ac}}$ ≤ 1 Oe) 3D magnetic fields is generally insufficient to power the hundred‐meter‐scale communication systems, despite the significant advantages that such systems are beneficial for long‐distance health monitoring of power infrastructure, and improve the safety of power plant workers.

Therefore, the main challenges of MME energy harvesting lie in ensuring that MME‐EHs can achieve multi‐directional resonance at 50/60 Hz under 3D magnetic fields, and obtain high power output to drive long‐distance LoRa communication. To overcome these challenges, this article proposes a gecko‐like MME‐EH, as shown in Figure 1. The gecko's torso is renowned for its flexibility and stability, especially in maintaining balance while climbing. Mimicking the gecko's limbs, the bionic structure can generate coordinated bending and twisting responses in 3D magnetic fields. Furthermore, this symmetric biomimetic design not only reduces clamping losses but also enables higher magnetic loads and larger beam deformation, thereby achieving the high‐efficiency collection of weak magnetic field energy. Through theoretical and experimental investigations, we demonstrate that the output power of the proposed harvester under the *x*‐, *y*‐, and *z*‐directional 1 Oe magnetic field is 12.8, 0.24, and 0.15 ${\mathrm{mW}}_{\mathrm{RMS}}$, respectively. Compared with existing studies, the proposed harvester represents the first demonstrated solution capable of achieving resonance and energy harvesting under the 3D 50/60 Hz magnetic fields, while simultaneously delivering superior power output and power density. Moreover, the experimental results confirm that the gecko‐like MME‐EH can successfully achieve complete energy autonomy for hundred‐meter‐scale LoRa communication. Ultimately, its practical feasibility for real‐time monitoring of the operational status of power infrastructure is demonstrated.

## 2 Results and Discussion

### 2.1 Design of the Gecko‐Like MME‐EH

**FIGURE 2:**
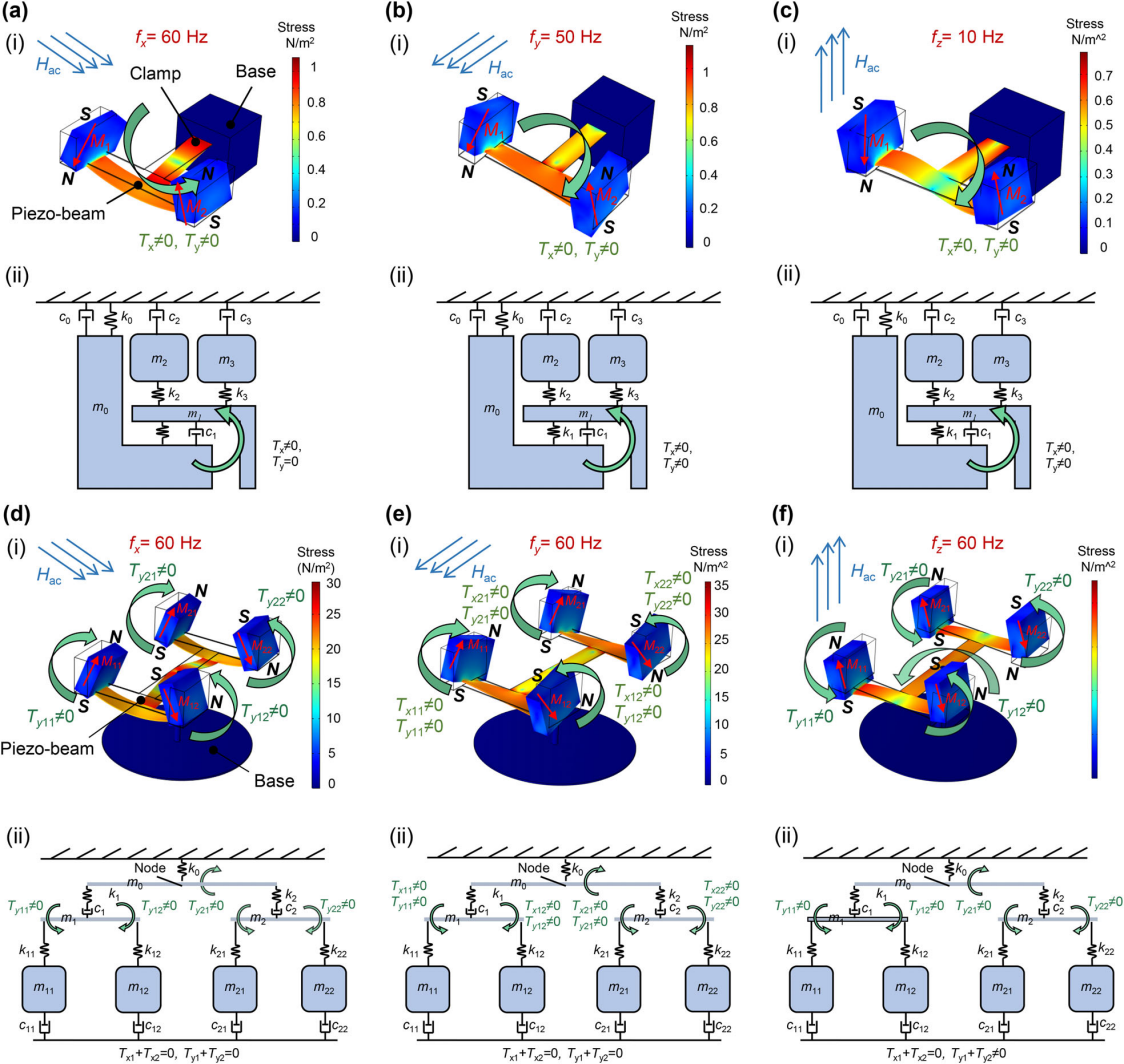
(a‐i), (b‐i), and (c‐i) Modal diagrams of the single‐sided MME‐EH when it is excited by magnetic fields in the *x*‐, *y*‐, and *z*‐directions, respectively. (a‐ii), (b‐ii), and (c‐ii) The corresponding equivalent spring‐mass system of the single‐sided MME‐EH, where $m_{0}$, $m_{1}$, $m_{2}$(=$m_{3})$, $c_{0}$, $c_{1}$, $c_{2}$(=$c_{3}$), $k_{0}$, $k_{1}$, and $k_{2}$(=$k_{3})$ are the equivalent mass, damping, and stiffness of the clamping part, transverse beams, and longitudinal beam, respectively, and $T_{x}$ and $T_{y}$ represent the magnetic torques around the x‐axis and y‐axis, respectively. (d‐i), (e‐i), and (f‐i) Modal diagrams of the gecko‐like MME‐EH when it is excited by magnetic fields in the *x*‐, *y*‐, and *z*‐directions, respectively. (d‐ii), (e‐ii), and (f‐ii) The corresponding equivalent spring‐mass system of the gecko‐like MME‐EH, where $m_{0}$, $m_{1}$(=$m_{2}$), $m_{11}$(=$m_{12}$=$m_{21}$=$m_{22}$), $c_{0}$, $c_{1}$(=$c_{2}$), $c_{11}$(=$c_{12}$=$c_{21}$=$c_{22}$), $k_{0}$, $k_{1}$(=$k_{2}$), and $k_{11}$(=$k_{12}$=$k_{21}$=$k_{22}$), are the equivalent mass, damping, and stiffness of the clamping part, transverse beams and longitudinal beams, respectively, and $T_{x11}\left(=T_{x12}=T_{x21}=T_{x22}\right)$ and $T_{y11}\left(=T_{y12}=T_{y21}=T_{y22}\right)$ represent the magnetic torques around the x‐axis and y‐axis, respectively.

Analogous to the gecko's torso, the gecko‐like MME‐EH is configured with longitudinal and transverse beams. Two pairs of permanent magnet (NdFeB) masses with specific magnetization directions and N/S polarities are mounted at both ends of the transverse beams. The magnets at the two ends of the cross beam have opposite polarities, while the magnets at the two ends of the longitudinal beam share the same polarity, thereby enabling the generation of the vibration mode induced by magnetic field excitations $H_{\text{ac}}$ as shown in Figure 2. The bending and twisting modes of the gecko‐like MME‐EH imitate the 3D movements of the gecko to ensure flexibility and maintain stability. The entire beam substrate is made of spring steel (ST), which has a higher mechanical quality factor $Q_{m}$ compared to a single copper (Cu) sheet. This enhances the MME coupling effect and leads to increased output power. Two thin polarized piezoelectric ceramic plates with dimensions of 60 mm (*L*) × 14 mm (*W*) × 0.2 mm (*T*) are glued to the surface of transverse beams with epoxy resin. The voltage or current generated by the piezoelectric plate due to the direct piezoelectric effect is referred to as the MME coupling effect. Other structural geometric parameters of the harvester are provided in Figure S1. In the experiment, the frequency of this gecko‐like energy harvester can be matched to 50/60 Hz, the standard frequency of alternating current, by adjusting the mass of the magnets at the free ends of the transverse beams. Note that $m_{0}$ and $m_{1}$ denote the equivalent mass of the clamping part and transverse beams, respectively. For the strongly clamped cantilever beam MME harvesters, they normally require a bulky clamping mass $m_{0}$ ($m_{0}>>m_{1}$) to maintain the stability of the system due to the asymmetry of the structure, as shown in Figure 2(a‐i,b‐i,c‐i). In contrast, the proposed centrally symmetric gecko‐like MME‐EH preserves its natural vibration node position located at the symmetry center, thereby minimizing clamping energy loss. Furthermore, compared with its single‐sided counterpart, the centrally symmetric structure can sustain higher magnetic load, resulting in higher magnetic torque and larger beam deformation. Consequently, the whole structure can achieve a higher level of output power at the same resonance frequency under magnetic field excitation in the x‐, *y*‐, and z‐directions.

When the gecko‐like MME‐EH is excited by magnetic fields in the x‐, y‐, and z‐directions, the following three vibration modes will be triggered: (1) in‐phase bending mode, (2) twist mode, and (3) anti‐phase bending mode. To benchmark against state‐of‐the‐art research on energy harvesting under multi‐directional magnetic fields of 60 Hz [41], the vibration modes of the gecko‐like MME‐EH are compared with those of its single‐sided T‐shaped counterpart, as shown in Figure 2. When the magnetic field excitation direction is along the x‐direction, the single‐sided MME‐EH produces strong clamping loss due to the magnetic torque around the x‐axis ($T_{x}\neq 0,T_{y}=0$), as shown in Figure 2(a‐i). Note that the figures only show the magnetic torque ($T_{x},T_{y}$) about the x‐axis and y‐axis, due to the fact that no magnetic torque about the z‐axis is generated in all cases. In fact, whether a synthetic torque at the clamping end $m_{0}$ exists or not depends on the vibration mode of the transverse and longitudinal beams. For the single‐sided MME‐EH, Figure 2(a‐ii) shows that the transverse beam with magnets ($m_{2}=m_{3},c_{2}=c_{3},k_{2}=k_{3}$) and the longitudinal beam vibrate in the in‐phase bending mode under the x‐direction excitation. As for the gecko‐like MME‐EH, when it is subjected to the magnetic field in the x‐direction, both the transverse and longitudinal beams undergo bending deformation due to the specific arrangement of four tip magnet masses ($M_{11}=M_{12}=M_{21}=M_{22}$), leading to the generated magnetic torque about the y‐axis (Here, $T_{y11}=T_{y12}=T_{y21}=T_{y22}$ are the magnet torques at the conjunction position between the transverse and longitudinal beams), as shown in Figure 2(d‐i). The vibrational modes of the gecko‐like MME‐EH can be described as the superposition of the bending modes of the transverse beams with four magnets ($m_{11}=m_{12}=m_{21}=m_{22},c_{11}=c_{12}=c_{21}=c_{22},k_{11}=k_{12}=k_{21}=k_{22}$) and the longitudinal beams ($m_{1}=m_{2},c_{1}=c_{2},k_{1}=k_{2}$), which is called the in‐phase bending mode. Thus, it is clearly observed that the net torque at its center mass $m_{0}$ is zero (i.e., $T_{x1}+T_{x2}=0,T_{y1}+T_{y2}=0$ with $T_{x1}$ and $T_{y1}$, and $T_{x2}$ and $T_{y2}$ respectively denote the torque of the half of the device symmetric about y‐axis), as shown in Figure 2(d‐ii). This indicates that the torques on both sides of the clamping part cancel each other out, reducing clamping energy loss at the center and enhancing the energy harvesting performance for the gecko‐like MME‐EH.

Similarly, when the external excitation $H_{\text{ac}}$ is along the *y*‐direction, the permanent magnets at both ends of the transverse beams can generate a pair of magnetic torques to excite the torsion mode of the gecko‐like MME‐EH, as shown in Figure 2(e‐i). Due to the structural symmetry, the magnetic torque ($T_{x1}+T_{x2}=0,T_{y1}+T_{y2}=0$) of the clamping part $m_{0}$ in the middle is also zero, as shown in Figure 2(e‐ii), leading to the low clamping loss. Specifically, despite that there exist non‐zero magnetic torques ($T_{x11}=T_{x12}=T_{x21}=T_{x22}$, $T_{y11}=T_{y12}=T_{y21}=T_{y22}$), their superimposition cancels out due to their opposite directions relative to the clamping position, leading to a zero net torque at the clamping part. Conversely, the torsional mode generated by the magnetic torque of the T‐type MME‐EH brings a non‐zero net torque ($T_{x}\neq 0,T_{y}\neq 0$) at the middle clamping part $m_{0}$, as shown in Figure 2(b‐i,b‐ii). Thus, the reduced clamping loss in the proposed gecko‐like energy harvester significantly improves its energy output under the y‐direction excitation at the same resonance frequency compared with its T‐shaped counterpart.

Unlike the two previous cases, when the external excitation $H_{\text{ac}}$ is applied along the *z*‐direction, a pair of magnetic torques arises from the permanent magnets ($T_{y11}=T_{y12}=T_{y21}=T_{y22}$) at the ends of the transverse beams in the gecko‐like MME‐EH, exciting an anti‐phase bending mode, as shown in Figure 2(f‐i,f‐ii). When operating in this anti‐phase bending mode, it can be observed that the deformation of the beam causes the center mass $m_{0}$ of two energy harvesters to oscillate about the *y*‐axis and the net torque of the clamping mass about the *y*‐axis appears to be non‐zero ($T_{y}\neq 0$). This oscillation can lead to structural instability and energy loss. However, compared with the torque ($T_{x}\neq 0,T_{y}\neq 0$) generated by the single‐sided MME‐EH at its center clamping mass $m_{0}$ Figure 2(c‐i,c‐ii), the net torque of the clamping mass about the *x*‐axis and thus the energy loss during operation in the proposed gecko‐like MME‐EH is still reduced ($T_{x1}+T_{x2}=0$). Furthermore, the centrally symmetric structure is more stable and permits more magnets with higher magnet torques and thus larger beam deformation. As a result, the output power of the gecko‐like MME‐EH under the *z*‐direction excitation is significantly improved during the actual operation. Movie S1 demonstrates three modes of the gecko‐like MME‐EH in the simulation.

In summary, compared with the classical cantilever and single‐sided T‐shaped MME‐EH, the gecko‐like MME‐EH with symmetrically distributed magnets exhibits reduced clamping loss, enhanced system stability, and higher magnet load capacity. These features enhance energy conversion efficiency and improve the mechanical quality factor, ultimately enabling higher output power generation. Thus, the proposed gecko‐like MME‐EH can not only achieve multi‐directional energy harvesting through coordinated twisting and bending operation modes, but also delivers a higher output power under low‐intensity magnetic fields ($H_{\text{ac}}$ < 1 Oe) with the same excitation frequency (50/60 Hz), contributing to the application prospects for stray magnetic field energy harvesting in the environment.

### 2.2 MME Coupling and Output Power of the Gecko‐Like MME‐EH

**FIGURE 3:**
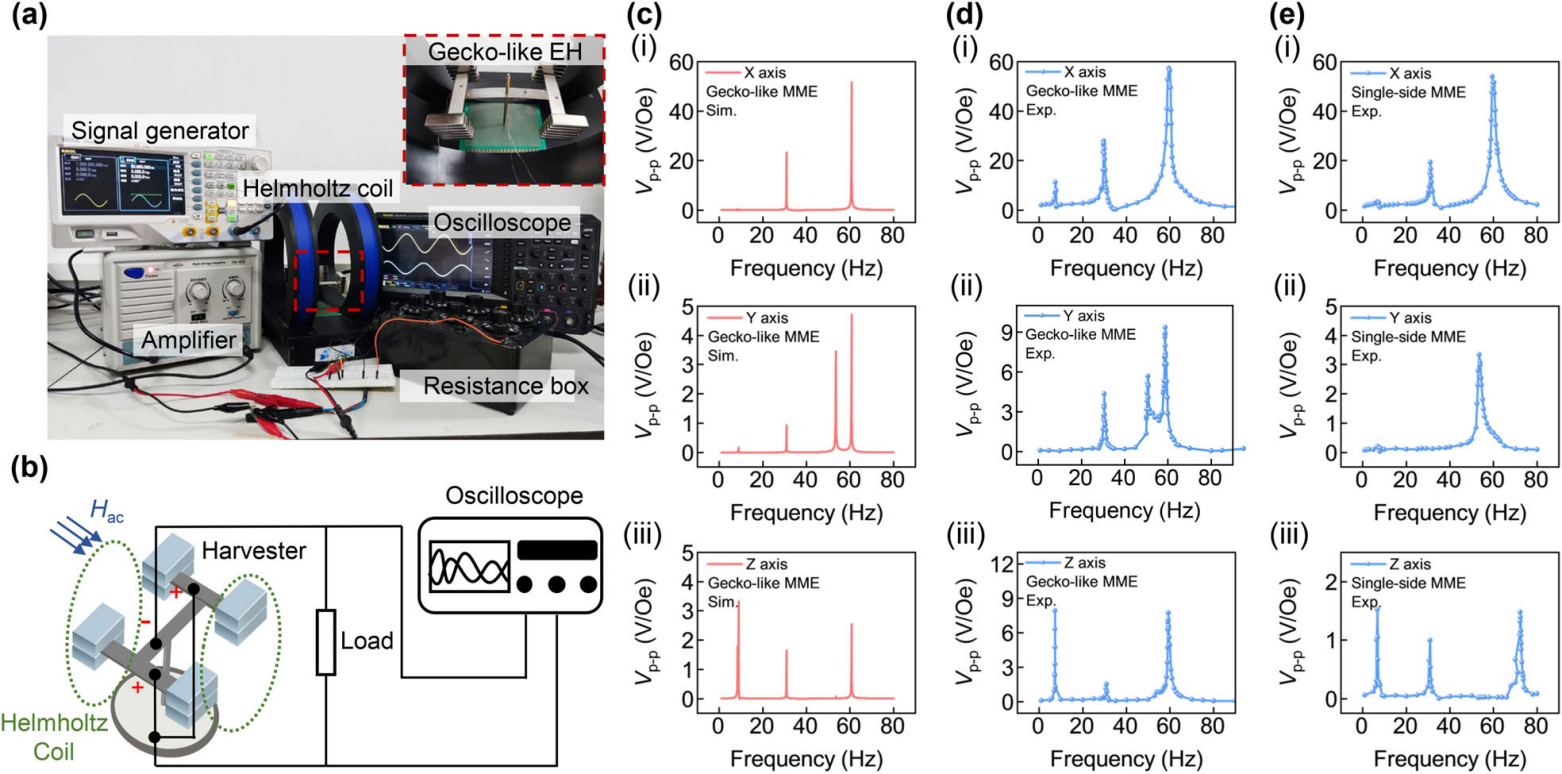
(a) Experimental setup. (b) Circuit configuration of the gecko‐like MME‐EH. (c‐i), (c‐ii), and (c‐iii) Simulation results of the frequency‐dependent voltage responses of the gecko‐like MME‐EH under the *x*‐, *y*‐, and *z*‐directional magnetic fields, respectively. (d‐i), (d‐ii), and (d‐iii) Experimental results of the frequency‐dependent voltage responses of the gecko‐like MME‐EH under the *x*‐, *y‐*, and *z*‐direction magnetic fields, respectively. (e‐i), (e‐ii), and (e‐iii) Experimental results of the frequency‐dependent voltage responses of the single‐sided MME‐EH under the *x*‐, *y*‐, and *z*‐directional magnetic fields, respectively.

For the multi‐directional MME energy harvesting, it is of significance to obtain high power output at the same resonance frequency (50/60 Hz) under magnetic fields from different directions. Through this design philosophy, sufficient energy can be captured from the stray magnetic field. In this section, finite element analysis (FEA) and experiments are used to elucidate the relationship between the output power and the excitation frequency for the proposed gecko‐like centrally symmetric structure in bending and torsion modes. Figure 3(a,b) shows the details of the prototype fabrication and experimental platform. In the experiment, a Helmholtz coil is employed to generate the magnetic field, the current of which is amplified by an amplifier. The gecko‐like energy harvester is placed inside the Helmholtz coil, and two piezoelectric sheets on the beam are connected in parallel, with this combined output connected to a resistor box. The voltage across the load resistance is recorded in real‐time using an oscilloscope. Note that the *x*‐, *y*‐, and *z*‐ magnetic fields are achieved by subsequently shifting the relative position between the harvester and the Helmholtz coil.

Figure 3(c‐i,c‐ii,c‐iii) plots the FEA results of the peak‐peak voltage response $V_{p}-p$ of the gecko‐like MME‐EH vs. the excitation frequency under a magnetic field $H_{\text{ac}}$ = 1 Oe along three directions. It can be observed that the resonance frequency peak positions obtained from the simulation are consistent with the experimental results shown in Figure 3(d‐i,d‐ii,d‐iii). When operating in the in‐phase bending mode Figure 3(d–i), the proposed gecko‐like MME‐EH exhibits a high voltage response peak of 57.4 V ${\mathrm{Oe}}^{-1}$ at the excitation frequency of 60 Hz. This in‐phase vibration mode is also consistent with that in Figure 2(d‐i). Besides, the first and second resonance peaks for the gecko‐like MME‐EH are observed at 7.5 and 29.6 Hz, with corresponding MME voltage responses of 11.3 and 27.9 V ${\mathrm{Oe}}^{-1}$, respectively. In addition, it can also be clearly seen from the experiment that the vibration modes at these resonance peaks are all located in Figure 2. The strong MME coupling in the proposed gecko‐like MME‐EH, evidenced by the high output voltage shown in the figure, stems from the carefully designed symmetrical structure that can withstand heavier magnets. This coupling results in a stronger magnetic torque acting on the piezoelectric composite cantilever, thereby yielding a higher energy output. At the resonance frequency of 60 Hz, the proposed gecko‐like MME‐EH demonstrates a 3 dB bandwidth of approximately 2.6 Hz, which represents a 53% improvement compared to a 1.7 Hz bandwidth in the T‐shaped MME‐EH in Ref. [41]. The corresponding ME coupling coefficient of the proposed energy harvester is calculated to be about 1014 V ${\mathrm{Oe}}^{-1}$ in this case, which is even comparable to those of MME‐EHs incorporating costly piezoelectric single crystals and highly textured Fe‐Ga alloys with higher piezoelectric constants [42, 43].

Figure 3(d‐ii) shows the dependency of the voltage response of the gecko‐like MME‐EH on the excitation frequency under $H_{\text{ac}}$ = 1 Oe along the *y*‐direction. A dominant resonance peak with an MME voltage response of 9.3 V ${\mathrm{Oe}}^{-1}$ occurs at the excitation frequency of 60 Hz, corresponding to the twist mode in Figure 2(e‐i). Two additional resonance peaks with MME voltage responses of 5.68 and 4.35 V ${\mathrm{Oe}}^{-1}$ appear at 50.8 and 30.5 Hz, respectively. Furthermore, when the magnetic field $H_{\text{ac}}$ = 1 Oe is applied along the *z*‐direction, the voltage response spectrum of the gecko‐like MME‐EH exhibits three peaks, as shown in Figure 3(d‐iii). The MME voltage response of 7.7 V ${\mathrm{Oe}}^{-1}$ for the gecko‐like MME‐EH is observed at the third‐order resonance frequency of 60 Hz, corresponding to an anti‐phase bending mode depicted in Figure 2(f–i). Additionally, the other two resonance peaks occur at 7.25 and 30.8 Hz, with corresponding MME voltage responses of 7.9 and 1.5 V ${\mathrm{Oe}}^{-1}$, respectively. The obtained results manifest that the proposed gecko‐like MME‐EH can achieve resonance and produce high output power at the same resonance frequency (60 Hz) under multi‐directional weak magnetic fields. This characteristic is attributed to the centrally symmetric gecko‐like structure and the distributed magnet configuration with tailored magnetization directions, which enable collaborative twisting and bending working modes. More importantly, the proposed MME‐EH has a strong 3D MME coupling response for enhanced output power.

To further validate the superiority of the gecko‐like MME‐EH, the voltage testing experiments for its single‐sided T‐shaped counterpart with the same material and geometric parameters of beams and magnets are conducted under the same magnetic intensity $H_{\text{ac}}$ = 1 Oe with different excitation frequencies, as shown in Figure 3(e‐i,e‐ii,e‐iii). Such single‐sided T‐shaped MME‐EH represents a state‐of‐the‐art design for harvesting multi‐directional stray magnetic field energy, as reported in Ref. [41]. The results show that the dominant excitation frequencies under magnetic fields from different directions are different from those of the gecko‐like MME‐EH. Specifically, the single‐sided T‐shaped MME‐EH is unable to produce peak power at the same excitation frequency of 60 Hz when exposed to multi‐directional magnetic fields. Comparing the gecko‐like MME‐EH with its single‐sided T‐shaped counterpart, it can be concluded that the gecko‐shaped MME‐EH has the advantage of resonating at the same excitation frequency when subjected to magnetic excitation from different directions. This is attributed to its fully symmetric structure, which ensures uniform mass distribution and stiffness properties in all directions, leading to consistent dynamic responses along all three axes. Therefore, changes in the excitation direction have minimal impact on its resonance frequency. In contrast, the asymmetric mass distribution of the unilateral structure results in variations in stiffness and inertia and inconsistent dynamic properties across different directions, which in turn affect its resonance frequency.

**FIGURE 4:**
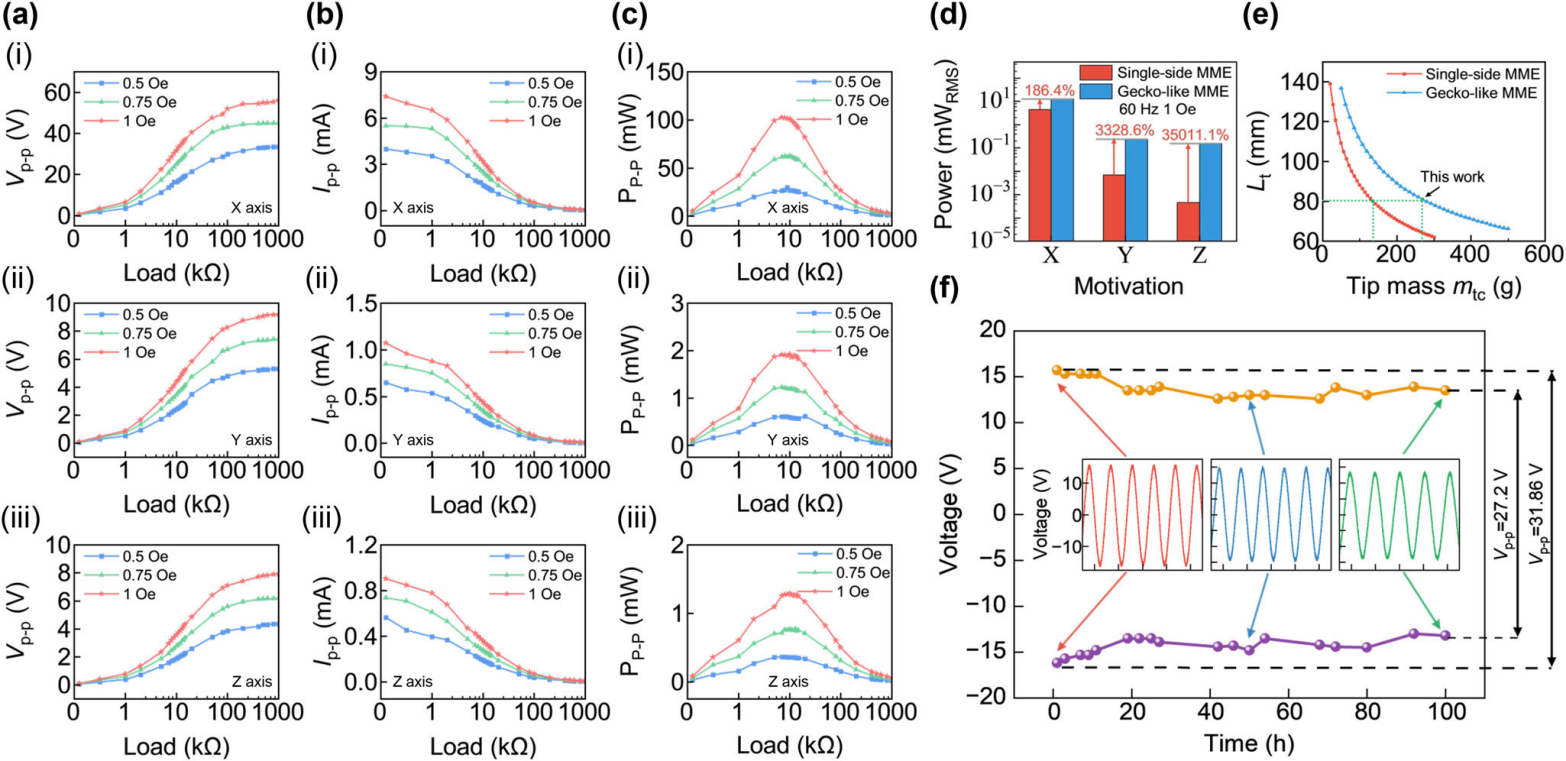
Experimental results: (a‐i), (b‐i), and (c‐i) output peak–peak voltage; (a‐ii), (b‐ii), and (c‐ii) output peak–peak current; (a‐iii), (b‐iii), and (c‐iii) output peak‐peak power of the proposed gecko‐like MME‐EH as a function of load resistance under varying $H_{\text{ac}}$ at their resonance frequencies along the *x*‐, *y*‐, and *z*‐directions, respectively. (d) Comparison of output power between the single‐sided MME‐EH and the gecko‐like MME‐EH under the *x*‐, *y*‐, and *z*‐directional magnetic fields. (e) Comparison of the transverse beam length $L_{t}$ as a function of magnet mass $m_{t}$ in the bending mode between the gecko‐like MME‐EH and single‐sided MME‐EH, each with a resonance frequency of 60 Hz. (f) Mechanical durability of the gecko‐like MME‐EH.

Figure 4 further presents the tridimensional energy harvesting performances of the gecko‐like MME‐EH in experiments. Figure 4(a‐i,b‐i,c‐i) illustrates the measured output peak–peak voltage, current, and power of the gecko‐like MME‐EH working in the in‐phase bending mode as a function of the load resistance $R_{L}$ subjected to different magnetic field intensities, respectively. In these tests, the magnetic excitation $H_{\text{ac}}$ along the *x*‐direction is applied at the resonance frequency of 60 Hz. It can be observed that the gecko‐like MME‐EH generates a maximum open‐circuit voltage of 56 V under an $H_{\text{ac}}$ field of 1 Oe at 60 Hz Figure 4(a‐i). As the magnetic field intensity decreases, the peak‐to‐peak voltage gradually decreases. At each magnetic field intensity, the output voltage gradually rises with increasing load resistance until it eventually saturates. The peak‐to‐peak current can be derived according to Ohm's law by extracting data from Figure 4(a‐i). The gecko‐like MME‐EH demonstrates a high peak‐to‐peak current of 7.4 mA under a weak magnetic field of 1 Oe, as illustrated in Figure 4(b‐i). While the output current increases with greater magnetic field intensity, it gradually declines until approaching zero with the increase of the load resistance for all magnetic field intensities. Furthermore, the peak‐to‐peak power $P_{p-p}$ can be calculated using the formula $P_{p-p}=\frac{V_{p-p}^{2}}{R_{L}}$, where $R_{L}$ and $V_{p-p}$ respectively denote the connected resistance and the peak‐to‐peak output voltage. As plotted in Figure 4(c‐i), the gecko‐like MME‐EH can generate a peak‐to‐peak output power of up to 102.7 mW and a normalized power of 12.8 ${\mathrm{mW}}_{\mathrm{RMS}}$ ${\mathrm{Oe}}^{-2}$ when connected to a load resistance of 9 kΩ under an excitation magnetic field of 1 Oe. Even at 0.5 Oe, it can also achieve an output power of 3.7 ${\mathrm{mW}}_{\mathrm{RMS}}$, demonstrating its suitability for low‐magnetic‐field‐intensity excitation scenarios in real‐world applications. Such energy level still surpasses that of MME‐EHs fabricated using expensive piezoelectric single crystals such as (PMN‐PZT single‐crystal macrofiber [20], PMN‐PZT single‐crystal macro fiber composite [20, 42, 43]), and tested in a stronger magnetic field ($H_{\text{ac}}$ > 1 Oe) [41].

Figure 4(a‐ii,b‐ii,c‐ii) illustrates the output performance of the bionic harvester in twist mode under the *y*‐directional magnetic field excitation. The change trends of voltage, current, and power output of the gecko‐like MME‐EH in this condition are similar to those observed under the varying *x*‐directional magnetic field intensities. For this case, the peak‐to‐peak output power reaches 1.9 mW (0.24 ${\mathrm{mW}}_{\mathrm{RMS}}$) under a magnetic field excitation of $H_{\text{ac}}$ = 1 Oe, as shown in Figure 4(c‐ii). Moreover, Figure 4(a‐iii,b‐iii,c‐iii) shows the output performances (i.e., voltage, current, and power) of the gecko‐like MME‐EH under the three $H_{\text{ac}}$ excitations along the *z*‐direction. Under $H_{\text{ac}}$ = 0.5 Oe, 0.75 Oe, and 1 Oe excitations, the maximum peak‐to‐peak output power is 0.36 mW (0.045 ${\mathrm{mW}}_{\mathrm{RMS}}$), 0.76 mW (0.095 ${\mathrm{mW}}_{\mathrm{RMS}}$), and 1.29 mW (0.16 ${\mathrm{mW}}_{\mathrm{RMS}}$), respectively. To clearly show the superiority of the proposed harvester, Figure 4(d) compares the output power of the proposed gecko‐like MME and its single‐sided T‐shaped MME counterpart at the target frequency of 60 Hz under $H_{\text{ac}}$ = 1 Oe excitations from different directions. To better illustrate the power increase in the *y*‐ and *z*‐directions, we apply a logarithmic transformation. Compared with its single‐sided counterpart, its power output of the gecko‐like structure is increased by 186.4%, 3328.6%, and 35011.1% under the *x*‐, *y*‐, and *z*‐directional magnetic field, respectively. Furthermore, the difference in the output power of the gecko‐like MME‐EH between the *y*‐ and *z*‐direction cases is only 51.27%, which is much lower than 1455.56 % observed in the single‐sided structure. Such a diminished difference arises from the fact that our proposed structure maintains stable resonance at 60 Hz under magnetic field excitation in all three directions, as shown in Figure 3(d‐i,d‐ii,d‐iii). In contrast, the single‐sided structure lacks full symmetry, preventing it from resonating at the same frequency under magnetic field excitations in the *y*‐ and *z*‐directions as shown in Figure 3(e‐ii,e‐iii). Additionally, the severe clamping loss in the single‐sided structure further exacerbates the power discrepancy. These experimental results fully demonstrate the capability of gecko‐like MME‐EH to capture energy from randomly oriented environmental magnetic fields near transformers or high voltage wires. Most importantly, the energy level of the gecko‐like MME‐EH under the multi‐directional magnetic field excitations is sufficient for powering high‐power‐consumption wireless sensor systems, such as the LoRa system, which will be demonstrated in Section 2.3.

The substantial increase in output power of the gecko‐like MME‐EH can be understood through the mechanical analysis. In the following, we consider the in‐phase bending mode as a representative. In this mode, the deformation of the longitudinal beam of the gecko‐like MME‐EH resembles that of a cantilever beam. As shown in the finite element model Figure 2(d‐ii), the system can be simplified and analyzed using a simply‐supported beam mechanics model. In this mechanical analysis, the single‐sided structure of the gecko‐like MME‐EH can be treated as a transverse simply‐supported beam connected in parallel with a longitudinal cantilever beam (Figure S2).

Due to the centrally symmetric design, the equivalent stiffness of the bionic harvester is twice that of the single‐sided structure. Additionally, the mechanical effect of the piezoelectric components on the overall structure can be ignored with the consideration of their relatively small geometrical dimensions compared to the single‐sided beam and magnets. Accordingly, the equivalent bending stiffness of the gecko‐like MME‐EH can be approximated by:

(1)

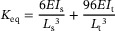

where $EI_{s}$ and $EI_{t}$ represent the equivalent bending stiffness of the longitudinal and transverse beams, respectively, and $L_{s}$ and $L_{t}$ denote their lengths. It is worth noting that, compared to the single‐sided MME‐EH, the enhanced equivalent bending stiffness indicates that the gecko‐like MME‐EH can support a heavier magnet mass at the target resonance frequency of 50/60 Hz, thus generating a larger magnetic torque and higher energy output. The equivalent mass of the gecko‐like MME‐EH can be calculated using the energy method as the sum of the equivalent mass of the transverse beams, the longitudinal beams, and the magnets, as follows:

(2) $$m_{\mathrm{eq}}=\frac{33}{70}m_{s}+m_{\mathrm{tb}}+0.0996m_{t}$$

where $m_{s}$ and $m_{\mathrm{tb}}$ represent the weights of the longitudinal and transverse beams, respectively. Also, $m_{t}$ is the total weight of the permanent magnets of the bionic MME‐EH. Based on these simplifications, the resonance frequency $f_{T}$ of the gecko‐like MME‐EH operating in the in‐phase bending mode can be expressed as follows:

(3) $$\begin{array}{cc} f_{T} & =\frac{1}{2\pi }\sqrt{\frac{K_{\mathrm{eq}}}{m_{\mathrm{eq}}}}\\ & =\frac{1}{2\pi }\sqrt{\frac{\left(420EI_{s}\frac{L_{t}^{3}}{L_{s}^{3}}+6720EI_{t}\right)}{\left(33m_{s}+70\rho AL_{t}+6.9720m_{t}\right)L_{t}^{3}}} \end{array}$$

where ρ and A are the density and cross‐section of the single‐sided sheet, respectively. At a fixed resonance frequency of $f_{T}$= 60 Hz, the dependence of the transverse beam length $L_{t}$ on the sum of the magnet mass $m_{t}$ is determined by solving Equation 3 (Figure 4(e)). Detailed derivation and the equation for the in‐plane bending mode are provided in Text S1. The two green points represent the calculated results of $L_{t}$ = 80 mm, $m_{t}$ = 136 g for the single‐sided MME‐EH and $L_{t}$ = 80 mm, $m_{t}$ = 270 g for the gecko‐like MME. These results align well with the experimental values of $L_{t}$= 80 mm, $m_{t}$ = 134 g, and $L_{t}$= 80 mm, $m_{t}$ = 268 g. Furthermore, compared to the single‐sided MME‐EH, the gecko‐like MME‐EH can accommodate heavier magnets, which achieves the greater magnetic torque in a stray magnetic field at a fixed operating frequency, thereby resulting in enhanced output power. Beyond the energy generation ability, as shown in Figure 4(f), the fatigue test of the proposed gecko‐like MME‐EH is performed. During operation, the output voltage is continuously monitored and recorded for 100 h under $H_{\text{ac}}$ = 0.5 Oe, after which a 14.6 % reduction is observed. Nevertheless, the gecko‐like MME‐EH has retained its structural integrity without any deformation or breakage. In particular, the PZT‐5H piezoelectric sheets and NdFeB magnets have remained undamage, even under prolonged exposure to environmental conditions. These results demonstrate the mechanical durability of the proposed harvester.

For clarification, Table 1 presents a comprehensive comparison between the proposed gecko‐like MME‐EH and various state‐of‐the‐art MME‐EHs in terms of the structural type, material, excitation frequency, magnetic field intensity, output power, and normalized power. It can be seen that the proposed gecko‐like MME‐EH leads in performance regarding multi‐directional magnetic field energy harvesting. Among the reviewed studies, only those reported in Refs. [40, 41] claim that their proposed MME‐EHs are capable of multi‐directional MME energy harvesting. Notably, the X‐shaped MME‐EH [40] is limited to the in‐plane magnetic field energy harvesting (i.e., along x‐ and y‐directions). Additionally, although the T‐shaped MME‐EH can achieve 3D magnetic field energy harvesting, its energy output along the y‐direction is ultra‐low. The above works indicate that the high‐efficiency energy harvesting under 3D magnetic fields is challenging. Compared with the T‐shaped MME [41] under the same magnetic field conditions ($H_{\text{ac}}$ = 1 Oe), the output power of the gecko‐like MME‐EH in the x‐, y‐, and z‐directions is increased by 2.12, 3.00, and 17.75 times, respectively. Moreover, under a lower magnetic field strength of 0.5 Oe, the gecko‐like MME‐EH can achieve an average power of up to 3.75 ${\mathrm{mW}}_{\mathrm{RMS}}$, demonstrating superior performance compared to existing MME energy harvesters.

**TABLE 1:** Comparison of magnetic‐field energy‐harvesting performances between the proposed gecko‐like MME‐EH and previously reported MME‐EHs.

Year	Structure	Materials	Excitation frequency [Hz]	Magnetic field [Oe]	Output power [${\mathrm{mW}}_{\mathrm{RMS}}$]	Normalized power [${\mathrm{mW}}_{\mathrm{RMS}}$ ${\mathrm{Oe}}^{-2}$]	Refs.
				1	12.8 (*x*)/0.24 (*y*)/0.15 (*z*)	12.8 (*x*)/0.24 (*y*)/0.15 (*z*)	
2025	Gecko‐like MME‐EH	PZT‐5H/ST	60	0.75	7.8 (*x*)/0.15 (*y*)/0.10 (*z*)	13.9 (*x*)/0.27 (*y*)/0.17 (*z*)	**This work**
				0.5	3.75 (*x*)/0.075 (*y*)/0.045 (*z*)	15 (*x*)/0.3 (*y*)/0.18 (*z*)	
2024	T‐shaped MME‐EH	PZT‐5H/ST	60	1	4.02 (*x*)/0.1(*y*)/0.008 (*z*)	4.02 (*x*)/0.1 (*y*)/0.008 (*z*)	[41]
2024	X‐shaped MME‐EH	PZT‐5H/ST	60	0.5	1.72 (*x*)/1.72 (*y*)	6.88 (*x*)/6.88 (*y*)	[40]
2023	Dragonfly‐wing‐like MME‐EH	PZT‐5H/Ti	50.5	1	4.45	4.45	[39]
2022	Tuning fork structured MME‐EH	PZT‐5H/ST	50.5	1	1.1	1.1	[38]
2022	Clamped‐clamped MME‐EH	PZT‐5H/Copper	50	0.5	0.37	1.48	[44]
2020	Cantilevered MME‐EH	PZT‐5A/Metglas	60	0.5	0.17	0.68	[45]
2019	Cantilevered MME‐EH	PZT‐5H/Ni	50	0.5	0.08	0.32	[33]

Table S2 compares the power density of the gecko‐like MME‐EH with previously reported MME‐EHs. It should be noted that the volume of magnets and beams is considered whereas the volume of clamping or supporting structure is not considered into the calculation of power density. It can be observed that the proposed gecko‐like MME‐EH still leads in terms of power density. Most importantly, the proposed harvester is the only harvester with comparable power densities under y‐ and z‐direction magnetic field excitation. The proposed device demonstrates significantly higher energy density, with an increase of up to fivefold along the z‐direction compared with Ref. [41]. Additionally, it is important to emphasize that the power density calculation of all MME devices in Table S2 excludes the bulky clamping end, which would otherwise lower the overall power density. Thus, if the clamping end is included for comparison, the gecko‐like MME exhibits a clearly higher power density, as its clamping is a single bolt with minimal mass.

In summary, compared with other MME‐EHs reported in Table 1 and Table S2, the proposed gecko‐like MME‐EH obviously exhibits superior multi‐directional energy harvesting performance. Qualitatively, the performance enhancement can be explained from two perspectives: (i) the gecko‐inspired design features a centrally symmetric structure enabling both bending and twisting modes when operation, which not only allows it to resonant at the same excitation frequency under multi‐directional magnetic fields but also reduces clamping losses, thus realizing high energy harvesting efficiency; (ii) the structure supports heavier magnets on the transverse beam, generating stronger magnetic torque and larger beam deformation under the weak magnetic field excitation, thereby enhancing its electromechanical coupling effect.

### 2.3 Self‐Powered LoRa Communication and Operational Status Monitoring System

Transformers, as core components of power infrastructure, are responsible for converting and transmitting electrical energy across voltage levels. The operating condition of transformers directly affects grid stability and safety, and any failures may cause large‐scale outages, equipment damage, or even fires, with serious economic and social consequences. Thus, real‐time, long‐term, continuous, and accurate monitoring of transformers is essential to guarantee their reliable operation and the security of the power grid. However, traditional inspection methods typically rely on manual operation, which exposes workers to significant safety risks from high voltage and high temperature. To alleviate this issue, based on the gecko‐like MME‐EH, we here develop a self‐powered LoRa communication to achieve remote operational status monitoring of the transformer.

**FIGURE 5:**
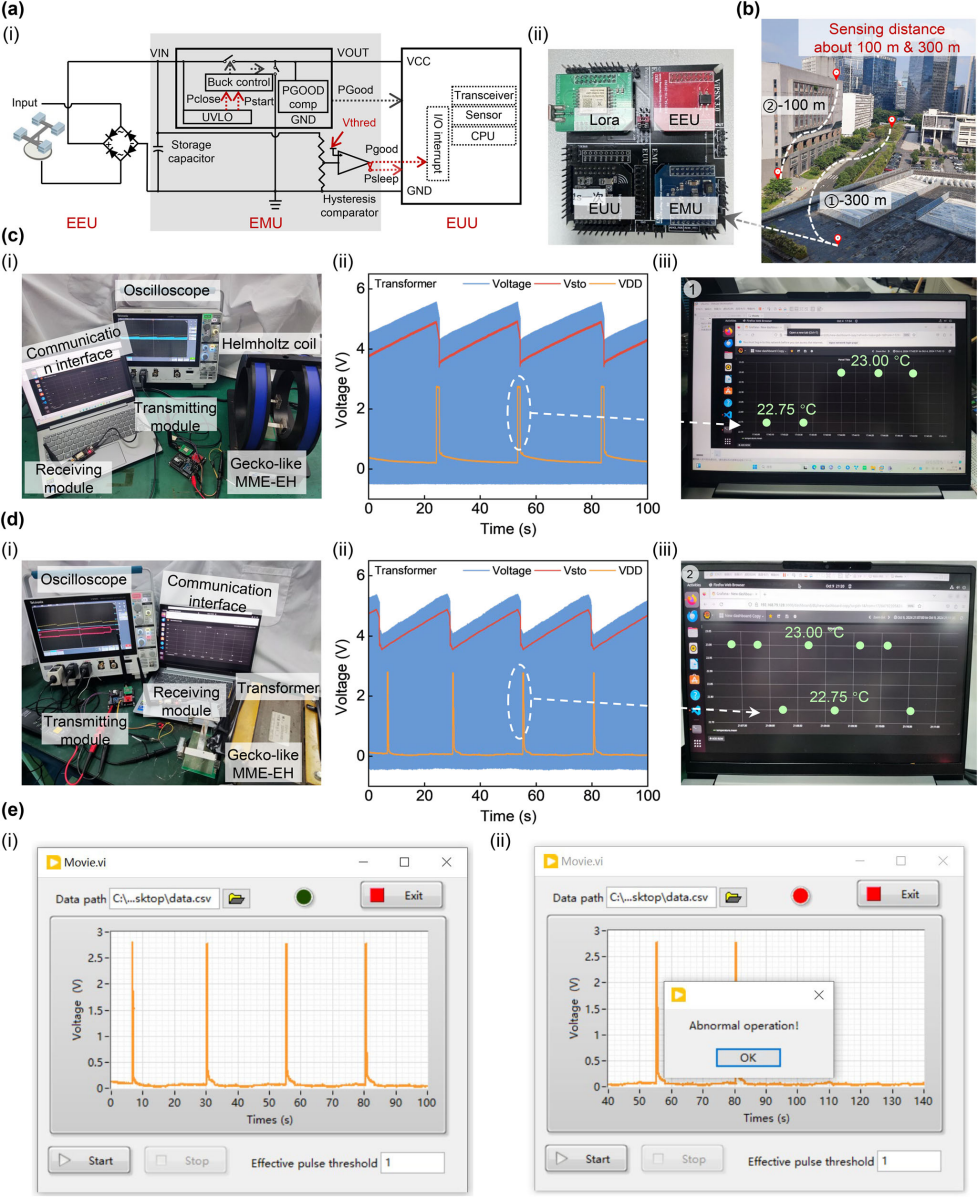
(a‐i) Circuit configuration of the self‐powered LoRa system integrated with the proposed MME‐EH. (a‐ii) Prototype of the ViPSN platform. (b) Sensing distances in outdoor and indoor environments. (c,d) Wireless sensing experiments utilizing (c) the Helmholtz coil and (d) the transformer: (i) experimental setup; (ii) time history of the voltage across the energy storage capacitor, the storage voltage (Vsto) of the capacitor, and the supply voltage (VDD) of the EUU in ViPSN; and (iii) temperature data from ViPSN received and displayed by the PC. (e): Screenshots of the power infrastructure anomaly early warning system: (i) normal operation and (ii) abnormal operation.

Figure S5 outlines the working principle of the monitoring system through a flow diagram. To achieve the self‐powered LoRa communication, an energy‐based vibration‐powered sensing node (ViPSN) is constructed, as shown in Figure 5(a‐i). ViPSN is a programmable platform that includes multiple modules: an energy enhancement unit (EEU), an energy management unit (EMU), an energy utilization unit (EUU), and a LoRa unit with a temperature sensor Figure 5(a‐ii). Initially, within the EEU, the AC signal from the gecko‐like multi‐directional energy harvester is converted into a DC signal via a full‐wave rectifier bridge. The EMU is responsible for voltage regulation and energy storage. In the EMU, the energy harvested by the gecko‐like multi‐directional harvester from stray magnetic fields is stored in a storage capacitor (6.8 mF), which can continuously provide a stable voltage supply to the energy utilization unit. The EMU adopts an enhanced design incorporating a buck converter, a storage capacitor, and an adjustable hysteretic comparator. It monitors the energy level through internal signals (Pstart, Pclose) and external signals (Pgood, Psleep) to control energy allocation and system state switching, thereby adapting to intermittent vibration environments and ensuring stable operation.

The EEU and EUU work in coordination: when the capacitor voltage reaches the startup threshold (5 V), the system initializes and enters a deep sleep mode to minimize power consumption. After stable energy accumulation raises the storage voltage to 5 V, the Pgood signal indicates sufficient energy, prompting the EUU to periodically perform temperature sensing and LoRa transmission. If the Psleep signal warns of insufficient energy, the system reverts to sleep mode to re‐accumulate energy. The EUU collaborates with the LoRa module to achieve sensor data acquisition and remote transmission. The LoRa unit, based on Chirp Spread Spectrum modulation technology, enables long‐range, anti‐interference communication in the Sub‐GHz frequency band and reduces power consumption through an intermittent operation mode. It exhibits high compatibility with the energy harvesting system, together forming a complete self‐powered wireless sensing system. Meanwhile, an oscilloscope is used to record the output voltage of the harvester, the storage voltage (Vsto), and the power supply voltage (VDD) of the EUU.

To demonstrate the capability of the gecko‐like MME‐EH for self‐powered LoRa communication, the in‐phase bending mode under the *x*‐direction magnetic field from a Helmholtz coil is utilized, as shown in Figure 5(c‐i). Specifically, the MME‐EH is placed inside a Helmholtz coil, and subjected to a weak alternating magnetic field ($H_{\text{ac}}$ = 0.5 Oe) with a frequency of 60 Hz, connected with the transmitting module of the ViPSN. And the receiving module of the ViPSN is placed at a distance of about 300 meters from the gecko‐like MME‐EH (see Movie S2), as shown in Figure 5b. Under the weak magnetic field of 0.5 Oe, a 6.8 mF capacitor reaches the $V_{\text{thred}}$ of 5 V after 23 s of charging Figure 5(c‐ii). Simultaneously, the voltage of the gecko‐like MME‐EH drops from approximately 5.5 V to about 4.5 V. The Vsto decreases from 5 V to about 3.7 V, while the VDD jumps from 0.4 to 2.8 V, indicating that the EUU successfully consumes the energy harvested from the gecko‐like MME‐EH. Furthermore, the signals transmitted by the EUU are successfully received by the PC, with five temperature signals oscillating between 22.75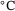 and 23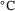 Figure 5(c‐iii). It is noteworthy that even after the Vsto drops, the capacitor voltage remains at 3.7 V, suggesting that a lower capacitor could be used to shorten the time interval between successive temperature signals. Unlike short‐range Bluetooth sensor systems, the LoRa system, whose communication distance can be extended up to 1 kilometers, requires higher energy consumption due to its long‐range capabilities. This experiment preliminarily confirms that the proposed harvester can successfully achieve the self‐powered LoRa communication in the Helmholtz coil testing environment.

To further evaluate the operational capability of the LoRa communication system in real‐world environments, the gecko‐like MME‐EH is then placed near a transformer Figure 5(d‐i). Since most power grid systems and transformers in China operate at 50 Hz, the resonance frequency of the harvester is adjusted to 50 Hz by adding magnets to both ends of the transverse beams. The magnetic field excitation along the x‐, y‐, and z‐directions is measured using a Gauss meter (Figure S3). It is found that the stray magnetic field strength of the transformer ($H_{\text{ac}}$ < 1 Oe) decreases cubically with increasing distance away from the transformer. Figure S4(a–c) respectively shows the output voltage waveform of the gecko‐like MME‐EH in the transformer experiments under magnetic field excitation along the three directions. The results show that the gecko‐like MME‐EH generates a maximum peak‐peak voltage of 23.6 V in the in‐phase bending mode. Even under magnetic field excitation along the y‐, and z‐directions, the gecko‐like MME‐EH can still produce peak–peak voltages of 3.8 and 2.4 V, respectively. Correspondingly, the average output power in the in‐phase bending mode reaches 3.14 ${\mathrm{mW}}_{\mathrm{RMS}}$, as shown in Figure S4(d). These results further demonstrate that the proposed gecko‐like MME‐EH possesses a distinct advantage in collecting stray magnetic field energy from multiple directions in real‐world applications.

As shown in Figure 5(d‐ii), in the transformer testing environment, the capacitor voltage rises from the baseline voltage ($V_{b}$ = 3.7 V) to the threshold voltage ($V_{\text{thred}}$= 5 V) after approximately 30 s of harvester operation, accumulating sufficient energy to power the temperature sensor. The measured temperature data are then transmitted to the PC via LoRa, with a transmission range spanning eight floors (approximately 100 m, see Movie S3), as shown in Figure 5(b,d‐iii). Since the signal attenuation is caused by the shielding effects of walls and floors, the transmission distance in this case is shorter than that observed in outdoor experiments. To derive the energy efficiency of the circuit, the energy stored in the capacitor $E_{\text{charge}}$ is calculated by:

(4) $$E_{\text{charge}}=\frac{1}{2}C_{p}\left(V_{\text{thred}}^{2}-V_{b}^{2}\right)=0.03845\;J$$

Thus, the actual energy delivered by the MME‐EH to the storage capacitor is 38.45 mJ. Subsequently, the capacitor charging power $P_{\text{charge}}$ can be expressed as follows:

(5) $$P_{\text{charge}}=\frac{E_{\text{charge}}}{T}=0.00128\;W_{\mathrm{RMS}}=1.28\;{\mathrm{mW}}_{\mathrm{RMS}}$$

where T represents the charging time. Therefore, with the energy harvester's power of 3.14 ${\mathrm{mW}}_{\mathrm{RMS}}$ harvested from the transformer, the power loss in the first‐stage rectifier bridge and the second‐stage energy management circuit is calculated to be 7.59 ${\mathrm{mW}}_{\mathrm{RMS}}$, leading to an overall energy efficiency of 14.4%. The primary sources of energy dissipation include the forward voltage drop loss in the rectifier bridge and thermal losses, as well as thermal dissipation caused by resistors, capacitors, and inductors within the EMU.

Based on the LoRa communication system, we propose a power infrastructure anomaly early warning system for remote and real‐time operational status monitoring of the transformer. For the sake of simplicity, the temperature sensor is utilized again in this situation. This system integrates data acquisition, logical judgment, anomaly warning, and human‐machine interface for alarm management. The logical flow diagram of the working principle is shown in Figure S5. When the transformer enters an abnormal operational status, the magnetic field would significantly decay or even vanish. Consequently, MME‐EH fails to operate efficiently, preventing the wireless sensor and communication unit in the LoRa system from being powered, leading to the delayed or lost signals displayed on the warning system. The system ensures the normal temporal characteristics of pulse signals through threshold comparison and time‐window detection. By comparing the real‐time input signal with a predefined “Effective pulse threshold,” it identifies valid pulses and records their triggering times. If no new valid pulse is detected within a predefined interval, the system determines an anomaly and issues an “Abnormal operation!” alert. The judging period of the abnormal operation event ΔT is related to the sampling frequency the sensor $T_{0}$. Figure S6 shows the diagram of the logical circuit developed in LabVIEW for the early warning system. The monitoring results for the transformer in the on and off states are shown in Figure 5(e‐i,e‐ii), respectively. When the transformer is turned off, the prompt window of the system displays an abnormal operation warning (see Movie S4). The demonstration validates the long‐distance health monitoring capability of the proposed gecko‐like MME‐EH due to its multi‐directional sensitivity to the magnetic field.

## 3 Conclusion

In conclusion, to achieve high‐performance multi‐directional magnetic field energy harvesting, this work develops a gecko‐like MME‐EH with coordinated bending and twisting modes at the resonance frequency of 50/60 Hz. The device is composed of a symmetrically arranged beam substrate, piezoelectric sheets, and magnets. This centrally symmetrical design effectively suppresses vibration energy loss, permits higher magnetic loads, and thus can generate larger beam deformation. Results demonstrate that even in a weak magnetic field environment with a frequency of 60 Hz and a magnetic field strength of only 1 Oe, the device can generate an average output power of up to 12.8 mW. The proposed device delivers a 17.75‐fold improvement in power output and a 5‐fold improvement in power density compared to the most advanced MME‐EHs reported previously. Under a lower magnetic field strength of 0.5 Oe, the device delivers an average power of up to 3.75 mW, representing the most superior performance compared with previous MME‐EHs reported in literature. Most importantly, this gecko‐like MME‐EH overcomes the issue faced by the existing multi‐directional MME‐EHs which could not achieve resonance at the same resonant frequency of 50/60 Hz in three different directions.

The high power output of the proposed gecko‐like MME‐EH under multi‐directional magnetic fields promotes its application in self‐powered long‐distance LoRa communication. The application tests show that the proposed MME‐EH can efficiently scavenge the energy from weak magnetic fields (< 1 Oe) near the Helmholtz coil or the transformer, and successfully powers the LoRa communications over distances of 300 meters outdoors and 100 meters indoors with time intervals of 23 and 30 s, respectively. Furthermore, by coupling the LoRa system with the anomaly early warning system, the unique operation status monitoring function of the proposed MME‐EH is experimentally demonstrated. This work suggests that the proposed design offers an efficient magnetic field energy harvesting strategy to support self‐powered long‐distance LoRa communication, and provides a novel strategy for the remote operation status monitoring of power infrastructure.

## 4 Methods

### 4.1 Fabrication and Characterization of the Gecko‐Like MME‐EH

The beam substrate was made of an ST plate [65Mn, spring steel (ST)] due to its excellent elasticity and corrosion resistance. PZT‐5H (Pante, Shenzhen, China) was selected as the piezoelectric material due to its relatively high piezoelectric coefficient and ease of integration. Each piezoelectric ceramic sheet, polarized along its thickness with a size of 60 mm (*L*) × 14 mm (*W*) × 0.2 mm (*T*), was attached to the base beam using epoxy resin (105/206, West System, Bay City, MI, USA) and cured at room temperature for 24 h. The piezoelectric sheets were connected in parallel using wires to optimize electrical output. Table S1 provides a comparison of the piezoelectric material used in this work and those previously reported in the literature. A small central hole was designed on the substrate for the installation of the beam, eliminating the need for an additional clamping mechanism in this centrally symmetric configuration. Magnetic masses (N52, NdFeB) were symmetrically mounted to the free ends of the beams, enabling them to produce pairs of magnetic torques or forces under an alternating magnetic field. A power amplifier (PINTECH, HA‐405) supplied power to the Helmholtz coils with a scaling factor of 152.27 Oe $A^{-1}$(MSO‐5104, RIGOL Technologies, Suzhou New District, China) to create a coaxial AC magnetic field. The output current of the power amplifier determined the strength of the AC magnetic field. An oscilloscope (RIGOL, MSO5104) was used to measure the induced voltage V across the piezoelectric plates under various AC magnetic fields, either through load $R_{L}$ or in an open‐loop circuit. The peak power P of the MME harvester was calculated using $P=\frac{V^{2}}{R_{L}}$, and the peak current I was determined according to Ohm's law.

### 4.2 Experimental Setup of LoRa Communication and Operation Status Monitoring Tests

A LoRa communication system was used to evaluate the energy generated by the proposed harvester in stray AC magnetic fields produced by Helmholtz coils or transformers. The magnetic field strength of the transformer is shown in Figure S3. Under $H_{ac}$ excitation, the induced output signal from the gecko‐like MME‐EH was converted into DC voltage via a custom full‐bridge rectifier and then stored in a 6.8 mF capacitor. An oscilloscope was used to capture the real‐time DC voltage across the storage capacitor, which powers the ViPSN platform for the LoRa communication. Environmental temperature data collected by the sensor was processed and displayed in real‐time on a laptop. As for the operation status monitoring test, the above testing environment was kept unchanged. Furthermore, the same LoRa communication system was used again and connected with the anomaly early warning system. The warning results of the power infrastructure were ultimately displayed on a laptop.

## Funding

This work was funded by National Natural Science Foundation of China (Grant Nos. 52575130, 52575129, 52375112), Guangdong Basic and Applied Basic Research Foundation, China (Grant No. 2026A1515010148), Shenzhen Natural Science Fund, China (Grant No. JCYJ20230808105206013), General Projects of Natural Science Foundation of Shanghai, China (Grant No. 24ZR1423200), Hong Kong Research Grants Council (Grant No. CUHK14211823), and The Chinese University of Hong Kong (Grant No. 4055178).

## Conflicts of Interest

None of the authors have a conflicts of interest to disclose.

## Supporting information


**Supporting File 1**: advs74974‐sup‐0001‐SuppMat.docx.

**Supporting File 2**: advs74974‐sup‐0002‐Data.zip.

**Supporting File 3**: advs74974‐sup‐0003‐MovieS1.mp4.

**Supporting File 4**: advs74974‐sup‐0004‐MovieS2.mp4.

**Supporting File 5**: advs74974‐sup‐0005‐MovieS3.mp4.

**Supporting File 6**: advs74974‐sup‐0006‐MovieS4.mp4.

## Data Availability

The data that support the findings of this study are available from the corresponding author upon reasonable request.
