# Supplementary material for: Gecko‐Like Multi‐Directional High‐Power Magneto–Mechano–Electric Energy Harvesters for Self‐Powered, Hundred‐Meter‐Scale LoRa Communication
Source: Adv Sci (Weinh). 2026 Mar 28;13(32):e74974. doi: 10.1002/advs.74974 (PMC13252662; doi:10.1002/advs.74974)
Supplement: Supplementary file 1 — Supporting File 1: advs74974‐sup‐0001‐SuppMat.docx. [file ADVS-13-e74974-s002.docx]

Supporting Information for

**Gecko-like multi-directional high-power magneto-mechano-electric energy harvesters for self-powered, hundred-meter-scale LoRa communication**

Shitong Fang, Haoxin Peng, Mingjing Cai^*^, Liuchao Jin, Yuelong Yu, Mianxin Xiao, Qingyang Xu, Jiacheng Hou, Degui Yu, Zhihui Lai, Xin Li, Shuxiang Dong^*^, Biao Wang^*^, Wei-Hsin Liao^*^

*Corresponding Authors: M. Cai (caimingjing@xidian.edu.cn); S. Dong ([sxdong@pku.edu.c](mailto:sxdong@pku.edu.c)n);

B. Wang ([biaowang6@shu.edu.cn](mailto:biaowang6@shu.edu.cn)); WH. Liao ([whliao@cuhk.edu.hk](mailto:whliao@cuhk.edu.hk)).

**This file includes:**

**Text S1 |** Calculations of the lumped effective mass and spring constant of the gecko-like MME-EH.

1. The geometric parameters of the gecko-like MME-EH used in this work.
2. The simplified mechanical model of the second-order bending mode in the gecko-like MME-EH.
3. Powering the LoRa wireless sensor and the communication system by the gecko-like MME-EH.
4. The corresponding voltage responses and output power of the gecko-like MME-EH in the transformer application tests.
5. Flow diagram showing the working principle and applications of the proposed device.
6. Diagram of the logical circuit developed in LabVIEW 2024 for the power infrastructure anomaly early warning system.
7. The comparison of the piezoelectric material used in this work and those reported in literature.
8. Comparison of the magnetic-field energy-harvesting performances in the proposed gecko-like MME-EH with those of the recently reported MME-EHs in literature.

**Additional materials for the article include the following:**

**Movies S1** to **S4**

Supplementary Text

**Text S1** | **Calculations of the lumped effective mass and spring constant of the gecko-like MME-EH**

To simplify the mechanical analysis, the vibration mode of the longitudinal beam on both sides of the support point can be treated as the first bending mode of a cantilever beam. Meanwhile, the transverse beam can be simplified as the first bending mode of a cantilever beam with two beam ends simply supported. Based on this, the displacement distribution function $w$ of the transverse beam in the second bending mode can be expressed as:

$w(x,t)=q(t)\sin\frac{\pi}{L_{t}}x$ (S1)

where *q*(*t*) represents the maximum displacement of the transverse beam, and *L_t_* is the length of the transverse beam. Its velocity function *v*(*x*, *t*) can be written as:

 (S2)

where the dot denotes the time derivative. *M_x_* represents the mass per unit length of the magnet. Thus, the kinetic energy of the transverse beam can be given by:

 (S3)

where *ρ* and *A* are the density and cross section of the ST sheet, respectively; the length of the magnet mass is taken as *L_t_*/8, and *m_tb_* is the mass of the transverse beam. Here, the transverse beam is simplified as uniform cross section.

Similarly, the equivalent mass of the longitudinal beam can be determined as $\frac{\text{33}}{\text{70}}\text{m}_{\text{s}}$. Thus, the equivalent mass of the gecko-like MME-EH is obtained as:

$m_{eq}=\frac{33}{70}m_{s}+m_{tb}+0.0996m_{t}$ (S4)

where *m_s_* is the mass of the longitudinal beam, and *m_t_* is the combined mass of the magnets attached at the two tip ends of the transverse beam. In them above simplified model, the transverse and longitudinal beams in the gecko-like MME-EH are considered to be connected in parallel. The equivalent stiffness of the parallel-connected transverse and longitudinal beams is:

$K_{\text{eq}}=K_{\text{s}}+K_{\text{t}}=\frac{6EI_{\text{s}}}{L_{\text{s}}^{3}}+\frac{96EI_{\text{t}}}{L_{\text{t}}^{3}}$ (S5)

where *E* represents the Young's modulus of the beam, and *I_s_* and *I_t_* denote the moments of inertia of the transverse and longitudinal beams, respectively.

The second-order resonant frequency of the gecko-like MME generator can be obtained using the following equation:

$f=\frac{1}{2\pi}\sqrt{\frac{k_{\text{eq }}}{m_{\text{eq }}}}=\frac{1}{2\pi}\sqrt{\frac{\left(420EI_{\text{s}}\frac{L_{\text{t}}^{3}}{L_{\text{s}}^{3}}+6720EI_{\text{t}}\right)}{\left(33m_{\text{s}}+70m_{\text{tb}}+6.9720m_{\text{t}}\right)L_{\text{t}}^{3}}}$ (S6)

Supplementary Figures

**Fig. S1 | The key parameters of the gecko-like MME-EH and the piezoceramic PZH-5H used in this work.**

**Fig. S2 | The simplified mechanical model of the second-order bending mode in the gecko-like MME-EH.**

**Fig. S3 | Powering the LoRa wireless sensor and the communication system by the gecko-like MME-EH.** When the gecko-like MME-EH is placed in three different orientations relative to the transformer, it is subjected to magnetic field excitations from the *x* (a), *y* (b), and *z* (c) directions. The magnetic field strength around the transformer is shown in (d).

**Fig. S4 | The corresponding voltage responses and output power of the gecko-like MME-EH in the transformer application tests.** Output voltage responses of the gecko-like MME-EH under the magnetic field excitation in the a) *x*, b) *y*, and c) *z* directions from the transformer. d) Output power of the gecko-like MMME-EH under the magnetic field excitation in the *x*-direction from the transformer.

**Fig. S5 | Flow diagram showing the working principle and applications of the proposed device.** Note that the dotted box shows the logical flow diagram to identify the occurrence of abnormal operation event. Note that $T_{0}$ is the sampling period of the sensor, and $\Delta T$ is the judging period of the abnormal operation event.

**Fig. S6 | Diagram of the logical circuit developed in LabVIEW 2024 for the power infrastructure anomaly early warning system.** All pixels are scanned by the logical circuit.

Supplementary Tables

1. The comparison of the piezoelectric material used in this work and those reported in literature.

|  | PZT-5H (used in this work) | PZT-5A | PZT-4 | PZT-8 | BaTiO_3_ | PVDF | PMN-33%PT | PZN-6%PT |
| --- | --- | --- | --- | --- | --- | --- | --- | --- |
| d_31_ (10^−12^ C/N) | −275 | −171 | −123 | −97 | −78 | −23 | −920 | −1400 |
| d_33_ | 593 | 374 | 289 | 225 | 149 | 33 | 2200 | 2400 |
| d_15_ | 741 | 584 | 496 | 330 |  |  |  |  |
| g_31_ (10^−3^ Vm/N) | −9.1 | −11.4 | −11.1 | −11 | 5 | 216 | −17.1 | 24.3 |
| g_33_ | 19.7 | 24.8 | 26.1 | 25.4 | 14.1 | 330 | 44 | 41.7 |
| g_15_ | 26.8 | 38.2 | 39.4 | 28.9 |  |  |  |  |
| *k_33_* | 0.75 | 0.71 | 0.7 | 0.64 | 0.48 | 0.15 | 0.93 | 0.9 |
| Mechanical *Q*_m_ | 65 | 75 | 500 | 1000 | 300 | 3–10 | 69 |  |
| Dielectric loss | 2% |  | 0.4% | 0.4% |  |  | 0.42% |  |
| Curie temperature (°C) | 193 | 365 | 328 | 300 | 115 | 100 | 145 | 100 |

1. Comparison of the magnetic-field energy-harvesting performances in the proposed gecko-like MME-EH with those of the recently reported MME-EHs in literature.

| Structure | Material | Magnetic field (Oe) | Output power  (mW_RMS_) | Normalized power  (mW_RMS_ Oe^-2^) | Power density  (mW_RMS_ Oe^-2^ cm^-3^) | Boundary  condition |
| --- | --- | --- | --- | --- | --- | --- |
| Gecko-like MME-EH (This work) | PZT-5H/ST | 1 | 12.8(*x*)/0.24(*y*)/0.15(*z*) | 12.8(*x*)/0.24(*y*)/0.15(*z*) | 0.272(*x*)/0.005(*y*)/0.003(*z*) | Simple  support |
|  |  | 0.75 | 7.8(*x*)/0.15(*y*)/0.10(*z*) | 13.9(*x*)/0.27(*y*)/0.17(*z*) | 0.295(*x*)/0.006(*y*)/0.004(*z*) |  |
|  |  | 0.5 | 3.75(*x*)/0.075(*y*)/0.045(*z*) | 15(*x*)/0.3(*y*)/0.18(*z*) | 0.318(*x*)/0.006(*y*)/0.003(*z*) |  |
| T-shaped MME [41] | PZT-5H/ST | 1 | 4.02(*x*)/0.1(y)/0.008(*z*) | 4.02(*x*)/0.1(*y*)/0.008(*z*) | 0.271(*x*)/0.006(*y*)/0.0005(*z*) | Bulky clamping |
| X-shaped MME [40] | PZT-5H/ST | 0.5 | 1.72(*x*)/1.72(*y*) | 6.88(*x*)/6.88(*y*) | 0.05(*x*)/0.05(*y*) | Simple  support |
| Dragonfly-wing-like MME [39] | PZT-5H/Ti | 1 | 4.45 | 4.45 | 0.262 | Simple  support |
| Tuning fork structured MME [38] | PZT-5H/ST | 1 | 1.1 | 1.1 | 0.164 | Bulky clamping |
| Clamped–Clamped MME [44] | PZT-5H/copper | 0.5 | 0.37 | 1.48 | 0.148 | Bulky clamping |
| Cantilevered MME [45] | PZT-5A/  Metglas | 0.5 | 0.17 | 0.68 | 0.24 | Bulky clamping |
| Cantilevered MME [33] | PZT-5H/Ni | 0.5 | 0.08 | 0.32 | 0.32 | Bulky clamping |

Additional materials for the article include the following:

**Movie** **S1** (.mp4 format). Coordinated bending and twisting modes at 60 Hz.

**Movie** **S2** (.mp4 format). Self-powered LoRa communication over 300 meters outdoors utilizing the Helmholtz coil as excitation.

**Movie** **S3** (.mp4 format). Self-powered LoRa communication over 100 meters indoors utilizing the transformer as excitation.

**Movie** **S4** (.mp4 format). Power infrastructure anomaly early warning system showing the abnormal operation condition.
